# Melanin: Production from Cheese Bacteria, Chemical Characterization, and Biological Activities

**DOI:** 10.3390/ijerph182010562

**Published:** 2021-10-09

**Authors:** Ana Rita Ferraz, Rita Pacheco, Pedro D. Vaz, Cristina S. Pintado, Lia Ascensão, Maria Luisa Serralheiro

**Affiliations:** 1 BioISI—Instituto de Biossistemas e Ciências Integrativas, Faculdade de Ciências, Universidade de Lisboa, Campo Grande 016, 1749-016 Lisboa, Portugal; ana3rita1@gmail.com (A.R.F.); rpacheco@deq.isel.ipl.pt (R.P.); 2 Área Departamental de Engenharia Química, Instituto Superior de Engenharia de Lisboa, Instituto Politécnico de Lisboa, Av. Conselheiro Emídio Navarro, 1959-007 Lisboa, Portugal; 3 Fundação Champalimaud, Av. Brasília, 1400-038 Lisboa, Portugal; pedro.vaz@fundacaochampalimaud.pt; 4 Escola Superior Agrária (ESA), Instituto Politécnico de Castelo Branco (IPCB), Quinta da Sra. de Mércoles, Apartado 119, 6001-909 Castelo Branco, Portugal; cpintado@ipcb.pt; 5 CERNAS/IPCB, Centro de Recursos Naturais, Ambiente e Sociedade/Instituto Politécnico de Castelo Branco, Av. Pedro Álvares Cabral 12, 6000-084 Castelo Branco, Portugal; 6 Centro para o Estudo do Ambiente e do Mar (CESAM), Faculdade de Ciências, Universidade de Lisboa, Campo Grande, 1749-016 Lisboa, Portugal; lmpsousa@fc.ul.pt; 7 Departamento de Química e Bioquímica, Faculdade de Ciências, Universidade de Lisboa, Campo Grande, 1749-016 Lisboa, Portugal

**Keywords:** bacterial products, *Pseudomonas* spp., eumelanin, antioxidant activity, acetylcholinesterase inhibition, cytotoxicity, cholesterol

## Abstract

Pigments are compounds of importance to several industries, for instance, the food industry, where they can be used as additives, color intensifiers, and antioxidants. As the current trend around the world is shifting to the use of eco-friendly commodities, demand for natural dyes is increasing. Melanins are pigments that are produced by several microorganisms. *Pseudomonas putida* ESACB 191, isolated from goat cheese rind, was described as a brown pigment producer. This strain produces a brown pigment via the synthetic Müeller-Hinton Broth. This brown compound was extracted, purified, analyzed by FTIR and mass spectrometry, and identified as eumelanin. The maximum productivity was 1.57 mg/L/h. The bioactivity of eumelanin was evaluated as the capacity for scavenging free radicals (antioxidant activity), EC_50_ 74.0 ± 0.2 μg/mL, and as an acetylcholinesterase inhibitor, with IC_50_ 575 ± 4 μg/mL. This bacterial eumelanin did not show cytotoxicity towards A375, HeLa Kyoto, HepG2, or Caco2 cell lines. The effect of melanin on cholesterol absorption and drug interaction was evaluated in order to understand the interaction of melanin present in the cheese rind when ingested by consumers. However, it had no effect either on cholesterol absorption through an intestinal simulated barrier formed by the Caco2 cell line or with the drug ezetimibe.

## 1. Introduction

The new awareness in humans of the need of non-organic synthesized compounds has created an enthusiasm for natural sources of colors. It is believed that natural dyes or dyes derived from natural and safe resources are non-toxic, non-carcinogenic, and biodegradable in nature. The dairy industry may be a good source of bacteria strains producing natural pigments. Brownish surface pigmentation is common in cheese made out of raw milk from sheep or goats [1]. It has been reported by cheese producers to cause severe economic losses, since it cannot enter the food market. *Pseudomonas* spp. have been isolated from the rind of brownish cheese in several environments, producing interesting extracellular molecules, such as enzymes [2] and biopigments [3]. Pigment production from biological non-pathogenic agents, such as those found in some food commodities, has attracted increased interests in recent years [3,4]. Nature produces many biopigments from various resources, including microorganisms, which are possible alternatives to synthetic dyes and pigments currently employed [5,6]. As the current trend around the world is shifting to the use of eco-friendly commodities, demand for natural dyes is increasing. The diverse biological roles of melanin in bacteria and fungi have been extensively reported in the literature [7]. Despite the fact that melanins are produced by a wide variety of microorganisms, they are not considered essential for its growth, being required in order to enhance the ability of the producing species to compete and survive under certain environmental conditions [8]. Generally, most microbial melanins are formed through the transformation of either tyrosine (DOPA-pathway) [7]. There are three types of melanins, i.e., eumelanins, pheomelanins, and allomelanins. Melanins tend to be either black or brown pigments, although other colors may occur [9]. Melanins derived from L-3,4-dihydroxyphenylalanine (L-DOPA), a tyrosine-derive amino acid, are referred to as eumelanins and are black or brown [9]. Reddish or yellow melanins that incorporate cysteine with L-DOPA are called pheomelanins [10]. Red-brown, water-soluble melanins formed from the catabolism of tyrosine via p-hydroxyphenylpyruvate (PHPPA) and homogentisic acid (HGA), are called pyomelanins [11,12]. 

Melanins are a unique class of natural pigments that can be considered functional biocompounds for multiple potential applications in industry, such as materials science, biomedicine, and cosmetics. The human nutrition pattern has changed in the last few years as there is an increasing concern with the origin of food, as well as its influence on human health. Alzheimer’s disease (AD) is considered one of the major public health problems worldwide. The cholinergic hypothesis was the rationale used to treat people with cognitive decline. It proposed that the neurotransmitter acetylcholine was in deficit in people suffering with AD. The main process to overcome this situation was to inhibit the enzyme responsible for the degradation of the neurotransmitter, acetylcholinesterase (AChE) [13]. AChE (E.C. 3.1.1.7) is an enzyme localized in the synaptic gaps and in neuromuscular junctions. These enzyme inhibitors increase the availability of the neurotransmitter, decreasing the cholinergic deficit and relieving the symptoms of AD patients [14]. Bacterial melanins have already been tested in number of animal models of neurodegeneration, including models of Parkinson’s disease [15], but the effect of melanin in Alzheimer’s disease (AD), as acetylcholinesterase (AChE) inhibitor, was not found in the bibliography.

Antioxidant activity in cells is another important biological activity, as it prevents Reactive Oxygen Species (ROS) formation and contributes to the reduction of inflammatory processes, which are the basis of several diseases [16]. 

Cardiovascular diseases (CVD) are the leading cause of mortality in Europe, responsible for over 3.9 million deaths a year, or 45% of all deaths [17]. One of the main causes of these diseases is the high cholesterol level circulating in the blood stream. Avoiding consumption of high cholesterol food is the first action to reduce hypercholesterolemia [18]. Melanin is a mixture of several phenolic compounds [19], and food products, as well as herbal infusions containing phenolic compounds, have been reported to have the capacity to reduce cholesterol absorption [16,20]; this topic will also be analyzed in the present work.

The main objective of the present study was to characterize and determine the bioactivity and cytotoxicity of a brown pigment produced by a strain of *Pseudomonas putida* isolated from a cheese industry.

## 2. Materials and Methods

### 2.1. Bacteria Source, Isolation, and Identification

*Pseudomonas putida* ESACB 191 belongs to the microbial culture collection of the Laboratory of Microbiology of Agrarian School of Polytechnic Institute of Castelo Branco, Portugal. It was isolated from goat’s cheese rind with brownish surface and was putatively identified using API 20NE (bioMérieux, Marcy l’Étoile, France).

### 2.2. Bacterial Growth and Melanin Production

For melanin production, 2.5 mL of a bacterial suspension (10^8^ CFU/mL) of *P. putida* ESACB 191 were inoculated in 22.5 mL of Müeller-Hinton Broth, MHB (OXOID, Basingstoke, Hampshire, England), containing 0.0 and 1.0, 2.5, 5.0, and 10.0 mg/mL of L-tyrosine (Acrós Organics, New Jersey, NJ, USA). The pH of the media was previously adjusted to 7.3 before autoclaving, and all the tests were carried out in triplicate. Bacterial growth was assessed on Nutrient Agar, NA (OXOID, Basingstoke, Hampshire, England), by the spread plate method, at the beginning and after 24, 48, and 72 h of incubation at 30 °C. After each incubation period, colonies were counted, and the colony forming units (CFU) were calculated per milliliter. 

### 2.3. Melanin Extraction, Purification, and Quantification

The extraction of pigment from the cell free supernatant was done by following the Seshagiri, Jalmi, and Bodke (2010) patent method [21], with minor changes. Briefly, cultures were centrifuged at 10,000× *g* for 10 min, the cell free supernatant was acidified with 1 N HCl to pH 1.5 and centrifuged again under the above conditions to precipitate the pigment, and supernatant was discarded. The brown pellet was washed 3 times with distilled water, centrifuged, and the pellet was collected and lyophilized. After the purification, the samples were weighed for quantification by dry weight of melanin produced by *P. putida* ESACB 191.

The quantification of melanin also was done through of total polyphenolic content method [19], using L-tyrosine as a standard. Total polyphenolic content was expressed as μg of L-tyrosine equivalents/mg of sample as the mean of three replicates.

### 2.4. Melanin Characterization

#### 2.4.1. Scanning Electron Microscopy (SEM)

The structure of the pigment isolated from *P. putida* ESACB 191 was investigated by SEM and compared with the morphology of commercial *Sepia officinalis* melanin (Sigma, St. Louis, MO, USA). The powder of the two samples was scattered carefully over a double-side carbon tape attached to the aluminum stub surfaces. Then, the material was sputter-coated with gold and the metalized samples observed on a JEOL 5200LV scanning electron microscope (JEOL Ltd., Tokyo, Japan) operating at an accelerating voltage of 20 kV.

#### 2.4.2. Fourier Transform IR Analysis (FTIR)

For FTIR studies, a Nicolet™ FTIR spectrometer from Thermo Electron Corporation was used with the DTGS Detector. The dried melanin (5 mg) was mixed with KBr (Sigma) and pelletized under mechanical pressure. The spectra were acquired in the spectral range of 400–4000 cm^−1^ with a resolution of 4 cm^−1^, accumulating 128 scans per spectrum. The spectrum was compared with the *S. officinalis* melanin.

#### 2.4.3. UHPLC-HRMS/MS Mass Spectrometry

The chromatographic analyzes to identify the compounds of the extracts was carried out by liquid chromatography-high resolution tandem mass spectrometry (LC-HRMS/MS) using an Elute OLE UHPLC system interfaced with a quadrupole time-of-flight (QqToF) Impact II mass spectrometer equipped with an electrospray source (ESI) (Bruker DaltoniK GmbH, Bremen, German). Chromatography separation was carried out on an Intensity Solo 2 1.8 µm C18 100 × 2.1 mm column (Bruker Daltonics, Bremen, Germany). The mobile phase, the elution conditions, and data acquisition by mass spectrometry as described in Reference [22]. The column and the sampler were maintained at 35 °C and 10 °C, respectively. The mass analysis was carried out in ESI negative and positive mode, the optimized parameters being: −3.5 kV and +4.0 kV; end plate offset, 500 V, nebulizer gas (N_2_) 2.0 bars; dry gas (N_2_), 8 L min-1; dry heater, 200 °C; collision cell energy was set to 5.0 eV. The internal calibration was performed with 250 mL H_2_O, 50 mL iPrOH, 750 µL acetic acid, 250 µL formic acid, and 0.5 mL 1 N NaOH solution on HPC mode. The acquired data were processed by DataAnalysis 4.1 software (Bruker Daltonik GmbH, Bremen, Germany). The identifications were carried out by taking into account the suggestions from the DataAnalysis^®^ program version 4.4 from BRUKER. The identification was based on the chemical formula proposed by DA program from Bruker (Bremen, Germany) and taking into account the mass fragmentation obtained with the equipment. Melanin from *Sepia officinalis* was used as a standard for natural melanins [23]. 

### 2.5. Biological Activities

#### 2.5.1. Antioxidant Activity

Antioxidant activity was measured using the 2,2-diphenyl-1-picrylhydrazyl (DPPH) method, as described by Falé et al., in 2009 [24]. The butylated hydroxytoluene (BHT) and ascorbic acid were used as standards and were bought from Sigma-Aldrich (Barcelona, Spain). 

All assays were performed in triplicate and are presented as mean and standard deviation associated to the measurements. In order to calculate the percentage of antioxidant activity, the following expression was used:

AA (%) = [(A control − A sample)/A control] × 100,

in which AA (%) corresponds to the percentage of antioxidant activity, A control refers to the absorbance of the control sample containing methanol, and A sample to the absorbance of the different extract solution. The concentration value to accomplish an extinction of absorption of 50% (EC_50_) was determined.

#### 2.5.2. Acetylcholinesterase Inhibitory Activity

Acetylcholinesterase (AChE) inhibitory activity was measured in triplicate, as described in Falé et al., in 2013. Acetylcholinesterase (AChE) inhibitory activity was measured in triplicate, as described in Falé et al., in 2013 [25]. Succinctly, to calculate the enzyme activity without inhibitor (100% activity), 325 µL of 50 mM Tris buffer pH 8, 100 µL of Milli-Q water, and 25 µL of AChE solution (1.33 U/mL) were mixed in a cuvette and incubated, at 25 °C, for 15 min. After 75 µL of AChI (0.33 mg/mL), 475 µL of DTNB (1.2 mg/mL of 50 mM Tris buffer pH 8 containing 0.1 M NaCl and 0.02 M MgCl_2_) were added, and the absorbance was read on the spectrophotometer (Schimadzu UV-160A, Matsuyama, Japan), at 405 nm, for 4 min, with 10-s intervals, and the initial velocity was calculated. The same procedure was carried out with the several samples, but, instead of adding 100 µL of Milli-Q water, the same volume of a solution of the various samples, at 1 mg/mL, was added. In this assay, the commercial standard used was galantamine. The reagents described were bought from Sigma-Aldrich (Barcelona, Spain).

#### 2.5.3. Cytotoxicity

The cytotoxicity assays were performed in several cell lines, such as A375 (ATCC^®^CRL-1619), HeLa Kyoto (CVCL_1922), HEPG2 (ATCC^®^ HB-8065), and Caco2 (ECACC_09042001).

All cell lines were cultured in DMEM, supplemented with 10% Fetal Bovine Serum (FBS), except Caco2 that were supplemented with 20% FBS; 100 U/mL penicillin, 100 U/mL streptomycin, and 2 mM L-glutamine at 37 °C in an atmosphere with 5% CO_2_; all chemical reagents were bought from Lonza (Verviers, Belgium). Cytotoxicity studies were performed using the MTT viability test [26]. Briefly, 50,000 cells/well were seeded in 96-well plates and incubated for 48 h at 37 °C in an atmosphere with 5% CO_2_. Then, the medium was replaced by several concentrations of the melanin solution (1 mg/mL) in 0.1% dimethyl sulfoxide (DMSO) and a control assay with the same media containing 0.1% DMSO. After incubation for 24 h in the same conditions, the media was replaced by 0.05 mg/mL MTT reagent in medium and incubated for 2 h. The MTT solution was removed and replaced with DMSO to mix the formazan crystals until dissolved, and the absorbance at 595 nm was registered against 630 nm. For each concentration of the sample, the percentage of viability was evaluated considering 100% viability in the control assay. The concentration of melanin solution that decreased 50% viability, IC_50_ values, was estimated for each cell line.

#### 2.5.4. Cholesterol Permeation Studies

Caco2 (ECACC_09042001), a human colorectal adenocarcinoma epithelial cell line was cultured as described in Falé et al., in 2013, and differentiated in transwell cells from Corning, Inc. (Corning, New York, NY, USA). To start the assays that were performed in triplicate, the cells were washed with Hanks′ balanced salt solution (HBSS), and 0.5 mL of the solutions tested contain: cholesterol (5 mM), ezetimibe (100 M), or melanin (1 mg/mL); in HBSS, they were applied into the transwell inserts (apical side of the cells). In the basolateral compartment, 1.5 mL of HBSS was added. After 6 h of incubation at 37 °C, 5% CO_2_, the solutions in both sides of the transwell compartment were collected and analyzed by HPLC.

### 2.6. Cholesterol Quantification by HPLC-DAD

Cholesterol was determined as described in Falé et al., in 2013 [27]. The HPLC–DAD analysis of the cholesterol quantification was carried out in an Elite LaChroms VWR Hitachi Liquid Chromatograph equipped with a Column Oven L-2300 and Diode Array Detector L-2455 (Tokyo, Japan). A column LiChroCARTs 250-4 LiChrosphers 100 RP-8 (5 mm) was used.

### 2.7. Data Analysis

The results were expressed as average ± standard deviation. The statistical difference between groups was calculated by one-way and two-way analysis of variance (ANOVA) was performed with α = 0.05, (95% confidence level) using the software Microsoft^®^ Excel 2016 (Microsoft Office 365, Redmond, Washington, DC, USA). 

## 3. Results

### 3.1. Melanin Bioproduction under the Influence of L-Tyrosine

**Figure 1:**
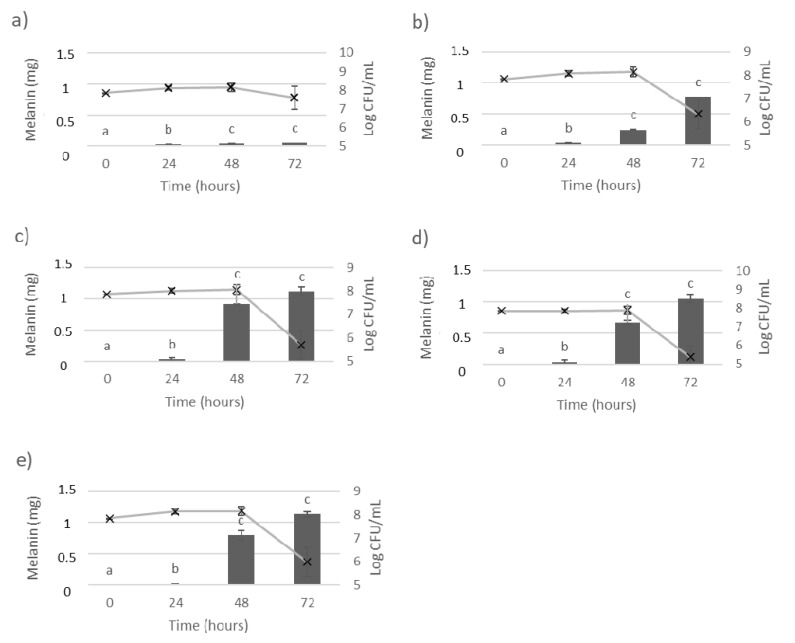
Influence of L-tyrosine concentration in *Pseudomonas putida* ESACB 191 growth and melanin bioproduction. The lines represent the *P. putida* ESACB 191 growth (Log CFU/mL) and bars represent the melanin bioproduction (mg), according to different amounts of L-tyrosine in the medium: (**a**) 0 mg/mL; (**b**) 1 mg/mL; (**c**) 2.5 mg/mL; (**d**) 5 mg/mL; (**e**) 10 mg/mL. The error bars values were calculated based on three replicates for each condition; different superscript letters (**a**–**c**) correspond to values in the same extraction condition that can be considered statistically different at confidence level of 95%.

The growth of *P. putida* ESACB 191 and bioproduction of melanin were studied by growing the bacterial culture in the medium Müller Hinton Broth without and with the precursor amino acid L-tyrosine, shown in Figure 1.

As shown in Figure 1a, it was found that, without amino acid, the control assay, *P. putida* ESACB 191, produced only 7.6% melanin when compared with Figure 1e. In Figure 1b–e, melanin production increases as the amount of the amino acid in the medium also increases, but bacterial growth is affected by increasing the concentration of L-tyrosine. A decrease in bacterial growth of about 2 logarithmic cycles was observed between 48 and 72 h of incubation. The production of melanin by *P. putida* ESACB 191 is dependent on the incubation time. It was verified for a 95% confidence level that it is from 48 h that melanin production increases significantly. 

**Figure 2:**
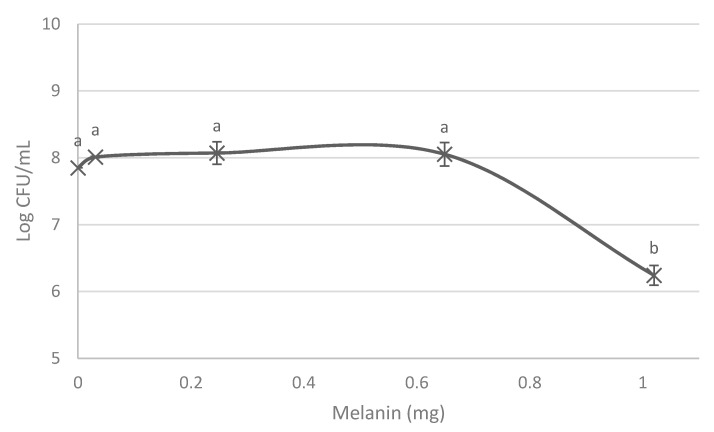
Effect of melanin production by *Pseudomonas putida* ESACB 191 in its growth and viability. The error bars values were calculated based on three replicates for each condition. (a, b) Different results at 95% confidence level.

Representing CFU/mL of *P. putida* ESACB 191 versus melanin produced, independently of the incubation period (Figure 2), it is possible to verify that the decrease of viability can be noticed after approximately 0.65 mg of melanin. The decrease in viability is so high, and the standard deviation of each value is so low, that we can infer a decrease in viability from 0.65 to 1 mg/mL of melanin. From 0.2 to 0.6, the decrease in viability is zero, and, from 0.6 to 1 mg/mL (the same 0.4 units), it is 22%.

**Figure 3:**
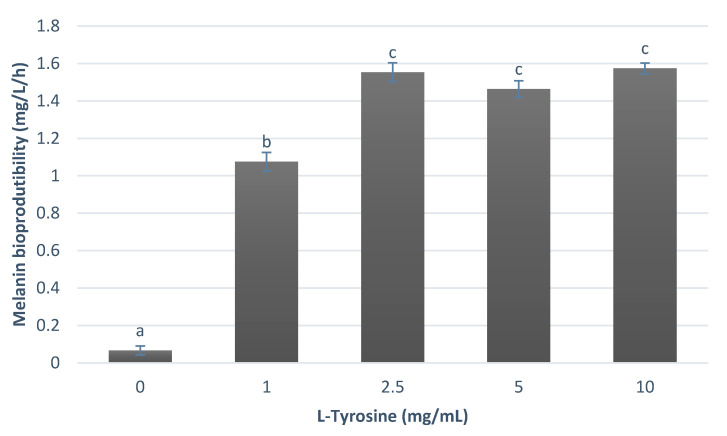
Melanin bioproductibility (mg/L/h) by *P. putida* ESACB 191, using different concentration of L-tyrosine in the culture medium. The error bars values were calculated based on three replicates for each condition; different superscript letters (a, b, c) correspond to values in the same quantification condition that can be considered statistically different 95% confidence level.

The effect of L-tyrosine concentration on melanin production can be seen in Figure 3. 

Considering the results presented, *P. putida* ESACB 191 has a maximum bioproduction of melanin of 1.57 mg/L/h when using 10 mg/mL of L-tyrosine. 

Figure 3 shows that melanin bioproduction depends on amino acid concentration. Maximum bioproduction was found to be achieved for concentrations greater than or equal to 2.5 mg/mL of amino acid for a 95% confidence level. Thus, taking into account that the concentrations of 2.5, 5, and 10 mg/mL of L-tyrosine do not show significant differences between them, Figure 3 also shows that, for the concentration of 2.5 mg/mL, a melanin bioproduction of about 1.55 mg/L/h was obtained, corresponding to a decrease of only 1.2%, compared to the maximum bioproduction of 1.57 mg/L/h at the concentration of 10 mg/mL. These results show that *P. putida* ESACB 191 has its maximum melanin bioproduction when the concentration of L-tyrosine is 2.5, meaning that we obtain a significant bioproduction using only a quarter of the maximum amino acid concentration, making the process more economical.

To confirm that the melanin produced by *P. putida* ESACB 191 detained compounds containing L-tyrosine derivatives, containing phenol groups, a quantification of phenolic compounds was carried out by determining the total phenolic content using a spectrophotometric method.

**Figure 4:**
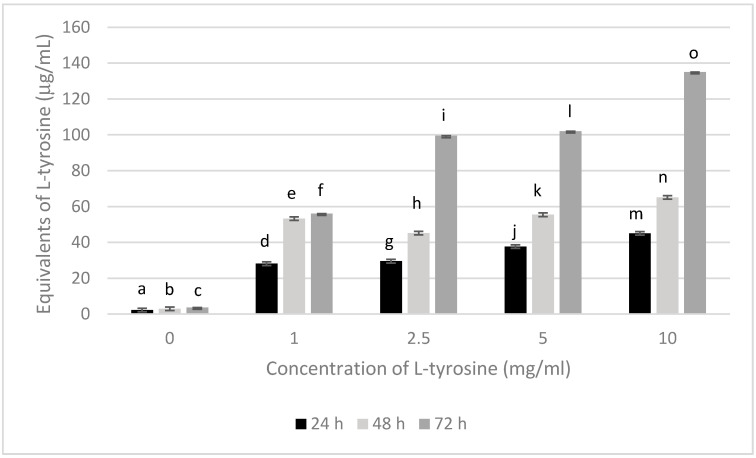
Total polyphenols present in melanin produced by *Pseudomonas putida* ESACB 191 with different concentrations of L-tyrosine. The error bars values were calculated based on three replicates for each condition. Different superscript letters (a–o) indicate different results at 95% confidence level, using a two-way ANOVA test.

Figure 4 shows the total polyphenols presents in melanin by *P. putida* ESACB 191 with different concentrations of L-tyrosine.

Figure 4 shows that the highest concentration of equivalents L-tyrosine were obtained when the bacteria were grown in the presence of the highest concentration of L-tyrosine. Melanin produced by *P. putida* ESACB 191 in the presence of the 10 mg/mL of the precursor L-tyrosine is about 32% more richer in phenolic compounds than the melanin produced with 5 mg/mL of L-tyrosine, for a confidence level of 95%. It is confirmed that the increase in melanin in mass (mg) means an increase in L-tyrosine-derived compounds containing the phenolic group, characteristic of black-to-brown subgroup of melanin formed by oxidative polymerization of tyrosine derivatives [7], considering the results at 72 h of incubation. Two-way ANOVA indicated an interaction at 95% confidence level between the amount of tyrosine and the time during which melanin was produced, with this interaction being more pronounced for 72 h. It can be seen from Figure 4, where the means are plotted, that the differences in the amount of melanin produced at 72 h is 360% higher than that produced at 24 h when comparing 10 and 1 mg/mL of tyrosine in the culture medium. The differences between the assays were also significant at 95% confidence level. The highest amount produced was obtained for 72 h using 10 mg/mL of trypsin. 

Thus, all other assays were performed with melanin produced by *P. putida* ESACB 191 after 72 h of growth in Müller Hinton Broth with 10 mg/mL of the precursor L-tyrosine.

### 3.2. Melanin Morphologic Analysis by SEM

**Figure 5:**
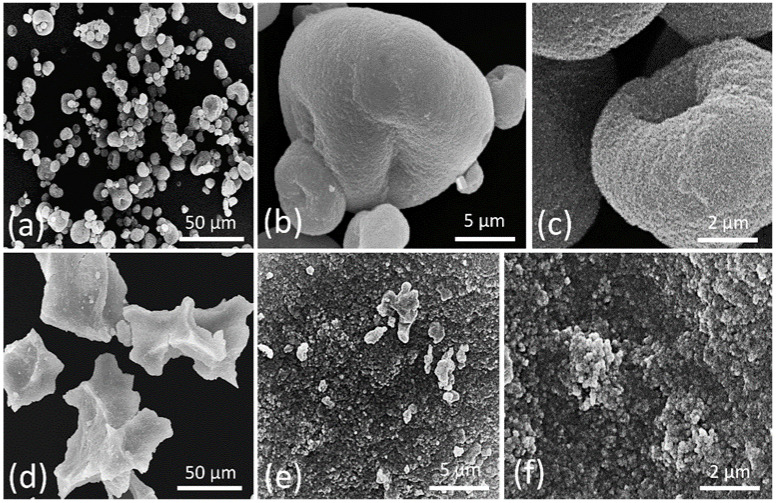
Scanning electron micrographs of *Sepia officinalis* melanin (**a**–**c**) and *Pseudomonas putida* ESACB 191 melanin (**d**–**f**). (**a**) General view, showing *S. officinalis* melanin aggregates with different sizes; (**b**) enlarged view of some melanin aggregates; (**c**) detail of the *S. officinalis* melanin aggregate surface; (**d**) lyophilized fragments of the extracted melanin; (**e**,**f**) enlarged views of melanin extracted fragments.

To compare the morphology of melanin produced by *P. putida* ESACB 191 used in the present study with a standard compound, melanin produced by *S. officinalis* was chosen. Both were observed by SEM (Figure 5). The micrographs showed that the microstructure of the two samples is different. 

*P. putida* ESACB 191 and *S. officinalis* melanin were observed by SEM (Figure 5) and showed that the microstructure of the two samples is different. SEM observations of the *S. officinalis* melanin revealed that it consists of multi-μm-sized aggregates of spherical granules, sometimes with collapsed profiles (Figure 5a–c). At higher magnifications, the surface of these granules is not smooth, it seems to be formed by closed packed particles (Figure 5c). In contrast, the bacterial melanin appears as a heterogeneous population of fragments, apparently amorphous without differentiable structures (Figure 5d). However, higher magnifications images of the fragment’s surfaces revealed that they are formed by aggregates of granules of spherical shape and different size (Figure 5e,f). The SEM imaging presented in the current study confirm what is reported in literature for *S. officinalis* melanin morphology [28]. Likewise, the SEM micrographs of the bacterial melanin are similar to those previously reported for other purified bacterial or synthetic melanin [29].

### 3.3. Melanin Structural Identification

To have an insight into the molecular structure of the compounds produced by *P. putida* ESACB 191, FTIR analysis and UHLC-HRMS/MS were performed. Both methodologies were applied to melanin from *P. putida* ESACB 191 and *S. officinalis*. 

#### 3.3.1. FTIR Analysis

**Figure 6:**
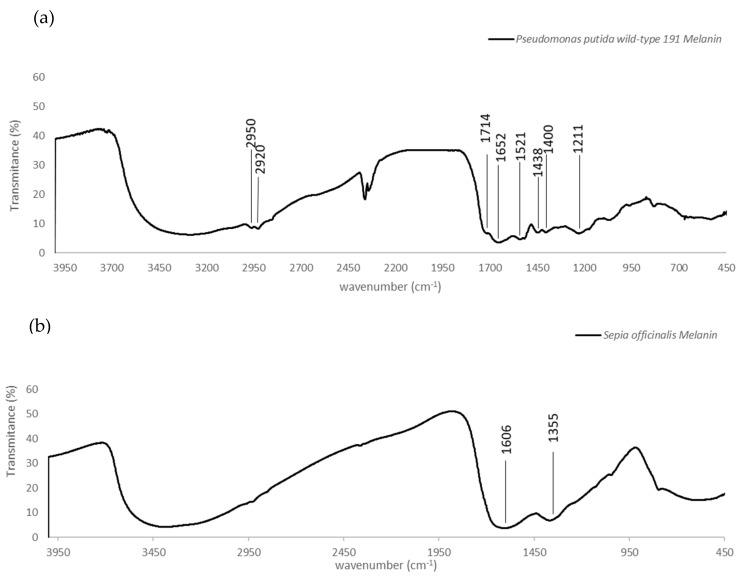
FTIR spectra in the range 4000–400 cm^−1^. (**a**) *Pseudomonas putida* ESACB 191 melanin. (**b**) *Sepia officinalis* melanin.

Figure 6 shows the FTIR of *P. putida* ESACB 191 (Figure 6a) and the *S. officinalis* melanins (Figure 6b). The ranges analyzed are indicated in Table 1 with the correspondent binding identification.

Figure 6 shows the FTIR of *P. putida* ESACB 191 (Figure 6a) and the *S. officinalis* melanins (Figure 6b). In Figure 6a, the 3700–3000 cm^−1^ range is due to O–H and N–H stretching modes. The profile of this broad and intense band arises from different O(N)–HO(N) hydrogen bonding geometries, which are probed by FTIR. At the lower end of this range (2950 and 2920 cm^−1^), there is a set of medium intensity bands which are assigned to C–H stretching vibrations. In Figure 6a, the appearance of bands in 1714, 1652, and 1521 cm^−1^ in the FTIR absorption spectrum may be related to both C=O and C=C stretching modes from carbonyl (possibly hydrogen bonded) and conjugated double bonds, respectively. The latter is due to the N–H bending modes which most probably are also involved in hydrogen bonding. In addition, this last pair of bands resembles the amide I/II bands from peptides. The absorptions detected at 1438 and 1400 cm^−1^ attributed to the asymmetric and symmetrical stretches of the –COO^−^ group. The bands at 1211 cm^−1^ may be related to the stretching vibrations of carboxylic acids/esters and the C–OH group [28]. Thus, FTIR spectra obtained from the pigment under study combine very well with FTIR absorption spectrum of indole-2-carboxylic acid reported in the literature and with those of some melanin produced by other bacteria [30]. 

#### 3.3.2. UHPLC-HRMS/MS Mass Spectrometry

**Figure 7:**
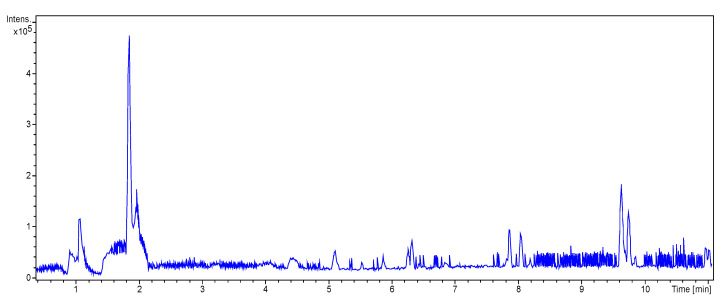
LC HRMS Chromatogram: melanin produced by *Pseudomonas putida* ESACB 191 in positive mode.

Liquid Chromatography (UHPLC) coupled with tandem high resolution mass spectrometry (HRSM/MS) was used to provide an insight into the structural characteristics of *P. putida* ESACB 191 melanin, as well as to determine molecular weight of the several compounds. As shown in Figure 7, the chromatogram indicated a complex structural diversity of this biopolymer. 

The chemical formula proposed by Data Analysis program™, Bruker, and the fragmentation pattern of each compound allowed the suggestion of the structures presented in Table 1. As can be seen, bacterial melanin consists of heterogeneous mixture of various molecular structures derived from tyrosine.

The bacterial melanin is a heterogeneous mixture of molecules, where the presence of tyrosine, 182.0808 (*m*/*z*), also suggested by the chemical formula C_9_H_12_NO_3_, can be detected. It can be concluded that the pigment under study is a mixture of tyrosine derivatives. The structures of the higher molecular weight compounds obtained were similar to those reported in melanin from marine origin [31].

### 3.4. Melanin Bioactivities

#### 3.4.1. AChE and Antioxidant Activities

The AChE inhibitory capacity of melanin, produced by *P. putida* ESACB 191, as well as its antioxidant activity, were studied, comparatively to standards (Table 2).

The bacterial melanin showed IC_50_ values for AChE inhibition of around 575 µg/mL, and the inhibitory activity is higher than that detected with melanin from *S. officinalis*. 

In fact, *P. putida* ESACB melanin showed better AChE inhibition than the commercial standard used for a 95% confidence level. This is a very interesting result for a compound of natural origin.

The *P. putida* ESACB 191 melanin showed a good antioxidant activity, EC_50_ 74 μg/mL, being approximately the twice the EC_50_ 39 μg/mL value with respect to the commercial standard BHT, for a confidence level of 95%. From a biotechnological point of view, this melanin of bacterial origin could be used as a powerful antioxidant in the food industry.

#### 3.4.2. Cytotoxicity

The cytotoxicity of the *P. putida* ESACB 191 and *S. officinalis* melanins were tested using the A375 and HeLa Kyoto cell lines, for both melanins, and using Caco2 and HepG2 cell lines for bacterial melanin, in order to calculate the cell viability and the IC_50_ values (Table 3). 

The cytotoxicity was tested in several cell lines, as shown in Table 3. The toxicity of the *P. putida* ESACB 191 and *S. officinalis* melanins was tested using three different concentrations (0.1, 0.5, and 1.0 mg/mL) of melanin to calculate the cell viability. The higher IC_50_ results than the concentrations tested were obtained from calibration curve with R^2^ 97. The melanins IC_50_ values obtained in cell lines tested were higher than the established limit of toxicity to human cell lines (0.1 mg/mL) [32]. Both melanins showed similar IC_50_ values, for a confidence level of 95%.

#### 3.4.3. Cholesterol In Vitro Intestinal Permeation

The non-cytotoxicity allowed the study of the effect of melanin on cholesterol permeability using Caco2 cell lines. After differentiating, 21 days in a transwell system, a monolayer, such as the intestinal barrier and expressing membrane transporter proteins responsible for cholesterol permeation, such as NPC1L1, is formed [33]. The determination of the permeability of cholesterol was expressed as the percentage of the compound that permeated from the apical to the basolateral compartment in the transwell system relatively to the control, shown Table 4. 

Table 4 shows that ezetimibe reduces cholesterol permeation to the basolateral comportment in basolateral in 87%, but melanin did not produce a significant reduction in cholesterol permeation for a confidence level of 95%. On the other side, the simultaneous administration to the cells of melanin with the drug ezetimibe did not reduce the cholesterol permeation in a substantial amount, which is a 10% reduction in the drug effect. 

## 4. Discussion

### 4.1. Production and Structural Analysis

Melanins are pigments that are produced by several microorganisms, such as mold [33], yeast [34], and bacteria [11]. Among the bacteria, *Pseudomonas* strains have also been referred to produce melanin [35], but *P. putida* had not been referred to as such before. *P. putida* ESACB 191 was isolated from a Portuguese dairy factory where, occasionally, browning of cheese rind appears, suggesting melanin production. This study is one example that melanin can be produced by *P. putida* ESACB 191. The effect of the amino acid L-tyrosine as precursor on melanin synthesis was evaluated. It could be seen that, by increasing the amino acid concentration in the culture medium, the amount of melanin also increased. It was found that the bioproduction of melanin by *P. putida* ESACB 191 in the presence of 1 mg/mL of L-tyrosine increased 93.5% compared to the control (0 mg/mL of L-tyrosine) at 72 h. Using concentrations equal to or higher than 2.5 mg/mL of the precursor amino acid, melanin bioproduction also increased by an average of 95% at 72 h. In addition, values greater than 0.65 mg of melanin decreased cell viability, in most cases, within 48 h, suggesting that the melanin produced presents toxicity to *P. putida* ESACB 191, which is a Gram-bacterium commonly described as more resistant to antibiotics. However, it was also found that melanin continued to be synthesized, even after a descent of 2 logarithmic cycles between 48 and 72 h of incubation. 

Although melanin decreased cell viability, the compounds went on being synthesized. This indicates that the enzyme responsible for its synthesis, probably tyrosinase, is an extracellular enzyme produced by *P. putida* ESACB 191. The most widespread pathways for melanin synthesis in bacteria involve melanin precursors derived from tyrosine transformations. This monohydroxylated compound is oxidized to yield dihydroxylated (diphenol) derivatives through reactions in which the amino group can be conserved [35], and this type of structure was detected in several compounds (Table 1). DOPA melanins are synthesized by tyrosinases. The tyrosinases (EC 1.14.18.1) are bifunctional copper-containing polyphenol oxidases that catalyze the hydroxylation of tyrosine to L-DOPA and its subsequent oxidation to o-dopaquinone [36]. This quinone suffers spontaneous cyclization to indole quinone, and, finally, this compound or its carboxylated form spontaneously polymerize to form brown to black DOPA melanins, also known as eumelanins [35]. The maximum melanin bioproductivity was 1.57 mg/L/h, while, in the study of Noura et al., in 2017, the maximum melanin bioproductivity by the bacteria *P. stutzeri* BTCZ10 was only 0.33 mg/L/h [37]. 

The chemical structure of melanin is still a matter of discussion. The chemical structure depends on the species synthesizing the compound or compounds. Melanin can be considered a mixture of several tyrosine derivatives [38]. The elemental analysis indicated that there was no sulfur in its composition, and it was an N-based melanin, a eumelanin. The compound produced by *S. officinalis* is also a eumelanin [21]. Therefore, this was chosen as a standard.

Scanning electron microscopy (SEM) is a powerful method for the morphological characterization and particle size distribution of different types of melanin. When comparing both melanins by scanning electron microscopy, it could be seen that there was a difference between both melanin that makes it foreseeable that the chemical structures will be different, due to different aggregations of the molecules leading to different macroscopic structures. Depending on the melanin source, the granule morphology and size range between 30–1000 nm and melanin granules are usually amorphous with irregular shape [19]. SEM observations of the *S. officinalis* melanin revealed that it consists of multi-μm-sized aggregates of spherical granules, sometimes with collapsed profiles (Figure 5c). At higher magnifications, the surface of these granules is not smooth, and it seems to be formed by closely packed particles (Figure 5c). In contrast, the bacterial melanin appears as a heterogeneous population of fragments, apparently amorphous without differentiable structures (Figure 5d). However, higher magnifications images of the fragment’s surfaces revealed that they are formed by aggregates of granules of spherical shape and different size (Figure 5e,f). The SEM imaging presented in the current study confirms what is reported in literature for *S. officinalis* melanin morphology. 

*P. putida* ESACB 191 melanin was also analyzed by FTIR analysis (Figure 6a) and compared with *S. officinalis* melanin (Figure 6b). The bands 2950 and 2920 cm^−1^ are often assigned to C–H stretching vibrations, which are typical of aromatic C–H groups from indole moieties, being similar to model compounds, such as indole itself and 5-hydroxy-indole [31], proven by Table 1. There are several bands indicating stretching mode vibration of C–O and C–C bonds, as well as N-H bending vibration modes. This is in accordance with the presence of N, C, and O in the chemical structure. In the fingerprint region, the absorptions at 1438 and 1400 cm^−1^ can be attributed to the asymmetric and symmetrical stretching of the –COO^−^ group, and the bands at 1211 cm^−1^ may be related to C–OH group stretching vibrations in carboxylic acids or esters [28]. Thus, the FTIR spectra obtained from the pigment under study was seen to be comparable with the FTIR absorption spectrum of indole-2-carboxylic acid reported in the literature and with those of some melanins produced by other bacteria [30]. FTIR spectroscopy indicates the presence of o-carbonyl groups and indazole groups meaning that compounds that might be derived from the amino acid tyrosine could be formed. 

The compounds identified seem to be derived from the amino acid L-tyrosine, what is in accordance with other melanins [33]. The bigger molecule had a molecular weight of 658.3334 Da and 654.2684 Da and had in its skeleton tyrosine derived residues. 

The existence of some phenolic units allowed the quantification of melanin mixture by total phenolic content using Folin-Ciocalteu reagent, and this was an easy and fast process of quantification for the components in this mixture. According to these results, it could be seen that the number of compounds containing phenolic groups increased after 72 h of bacterial growth, which is in accordance with the value obtained by mass quantification.

### 4.2. Bioactivities

The inhibition of AChE is used clinically to improve the quality of life in Alzheimer Disease (AD) patients, as well as in severe intestinal disorders [39]. Oxidative stress inside the cell can be the cause of several diseases, including neurodegenerative diseases and cardiovascular disorders. In the last few years, the recommendation for the use of natural antioxidants that can be obtained through food has grown, helping to prevent the formation of oxidative stress inside the cells being a generic rule [40]. 

When analyzing the melanin as AChE inhibitor, an IC_50_ values of 575 μg/mL was determined. Although this value indicates less activity than the drug galantamine 0.22 μg/mL, it has a much higher activity than herbal infusions with IC_50_ between 0.62 and 1.79 mg dry extract/mL, for instance [41]. A possible explanation for the inhibitory activity detected with melanin mixture lies in the fact that the compounds have aromatic rings in their structure, which allow the formation of the π–π (stacking) between these aromatic residue rings of the catalytic gorge of the AChE active site. The presence of aromatic rings makes the electronic cloud associated with the molecule larger, and, consequently, the probability of multiple enzyme-ligand interactions will also be greater [42]. 

The antioxidant activity relates to the *P. putida* ESACB 191, shown in Table 2. The *P. putida* ESACB 191 melanin showed good antioxidant activity, with EC_50_ 74 μg/mL being approximately twice that of the BHT control, EC_50_ 39 μg/mL. The good antioxidant activity of melanin can be explained through the fact that this melanin is a mixture of different compounds with hydroxyl and aromatics groups in their structure that can easily be stabilized by resonance upon the leaving of a hydrogen to the radical DPPH.

When studying the melanin mixture cytotoxicity using 4 different cell lines, it could be seen that there is no cytotoxicity toward all these cells, as all the cell lines showed an IC_50_ higher than 0.1 mg/mL, the established limit of toxicity to human cell lines for mixture of natural compounds [32]. The values were identical to those determined with *S. officinalis* melanin. 

This non-cytotoxicity allowed the study of the effect on cholesterol permeability and possible interaction with the drug ezetimibe by using 0.5 mg/mL of melanin mixture into contact with Caco2 cell monolayer, after establishing differentiation during 21 days in a transwell system, simulating, this way, the intestinal barrier. Ezetimibe monotherapy is recommended as an option for treating patients with primary hypercholesterolemia (heterozygous familial and non-familial) for whom statins were contraindicated or were statin-intolerant [43]. Ezetimibe showed a powerful reduction of cholesterol permeation, with about 87% of decrease. Melanin from *P. putida* ESACB 191 did not produce a significant reduction in cholesterol permeation, meaning that the compounds cannot chelate cholesterol nor block the membrane protein cholesterol transporter NPC1L1. On the other side, the simultaneous administration of melanin with the drug ezetimibe does not interfere with the drug activity. To the best of our knowledge, there are no reports of the effect of melanin on the cholesterol intestinal permeation, and results presented in this work are an indication that there will be no interaction between the samples and the cholesterol reduction-absorption drug, for a confidence level of 95%. 

## 5. Conclusions

The melanin studied has microbial origin and was isolated from cheese rind. This study allowed the isolation of the bacteria producing this pigment and the evaluation of its bioactivities, as well as its chemical characterization and identification. The melanin produced by *P. putida* ESACB 191 is a eumelanin with chemical structure DOPA, and some other compounds derived from this tyrosine, and the highest molecular weight detected was 658.3344 Da. The maximum bioproduction of melanin was 1.57 mg/L/h. 

The melanin from *P. putida* ESACB 191 was found to have interesting bioactivities from the biotechnological point of view, namely the high acetylcholinesterase inhibiting activity and antioxidant activity, whose results showed double the bioactivities of the commercial standards used in each assay. This naturally occurring melanin showed no cytotoxicity for the cell lines tested and no influence on intestinal cholesterol permeation simulated by monolayer Caco2 cells. Due to the tested bioactivities, several applications for this compound can be envisaged, either as food colorant, food antioxidant, or even in the cosmetic and pharmaceutical industry. 

## Figures and Tables

**Table 1:** Characterization of compounds present in *Pseudomonas putida* ESACB 191 melanin by UHPLC (+) HRMS/MS.

RT (Min)	Accuracy [M + H]^+^ (*m*/*z*)	Intensity	MS^2^ Fragment Ions [*m*/*z*, Attribution, (Intensity %)]	Proposed Compound	Chemical Structure
1.9	182.0808	5.3 × 10^5^	91.05 (100); 165.07 (58%); 136.07 (60.9); 123.04 (45.7); 119.04 (36.1); 65.03 (32.5); 95.04 (27.4); 77.03 (21.2)	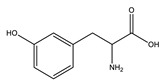	C_9_H_12_NO_3_ Tyrosine Error = −0.2
2.4	195.1128	4.6 × 10^4^	195.11 (100%); 167.00 (6.8%); 125.07 (11%); 98.06 (9%) 70.06 (36%)	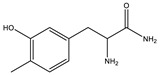	C_10_H_14_N_2_O_2_ Error = −1
5.7	231.1124	4.6 × 10^4^	214.08 (15%); 188.07 (17%); 158.09 (100%); 143.07 (56%); 130.06 (22%); 115.05 (11%); 74.02 (20%)	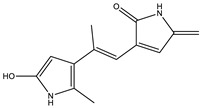	C_13_H_14_N_2_O_2_ Error = 4
5.9	227.0818	8.5 × 10^4^	199.08 (100%); 181.07 (40%); 154.06 (40%); 128.05 (10%)	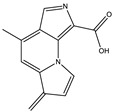	C_13_H_10_N_2_O_2_ Error = 1.1
6.3	361.1051	9.5 × 10^4^	315.09 (65%); 297.08 (11%); 269.09 (27%); 255.06 (53%); 237.05 (26%); 209.05 (31%); 191.04 (16%); 181.06 (16%); 163.05 (39%); 135.05 (26%); 107.04 (100%)	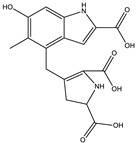	C_17_H_16_N_2_O_7_ Error = 1.5
6.9	245.1307	4.1 × 10^4^	154.07 (23%); 120.08 (41%); 98.06 (11%)	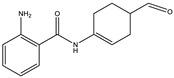	C_14_H_16_N_2_O_2_ Error = 2
6.9	659.3416	1.1 × 10^4^	614.17 (2%); 609.66 (2%); 521.70 (2%); 491.24 (6%); 344.18 (28%); 227.10 (42%); 217.13 (9%); 199.10 (37%); 182.13 (3%); 169.09 (35%)	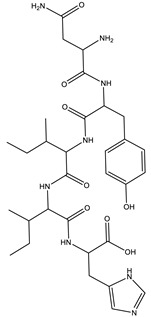	C_33_H_42_N_10_O_5_ Error = 0
7.9	318.0970	7.1 × 10^4^	272.09 (20%); 165.05 (27%); 147.04 (15%); 138.03 (11%); 123.04 (29%)	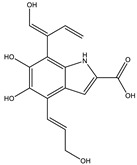	C_16_H_15_NO_6_ Error = 0
8.0	558.1058	7.1 × 10^4^	512.10 (94%); 498.08 (20%); 452.07 (48%); 346.03 (42%); 308.04 (81%); 289.03 (46%); 107.04 (20%)	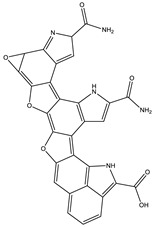	C_30_H_15_N_5_O_7_ Error = −2.9
8.0	580.0888	5.5 × 10^3^	536.10 (8%); 324.08 (15%); 276.02 (11%); 235.00 (21%); 107.04 (13%)	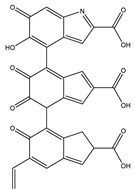	C_31_H_17_NO_11_ Error = −0.8
10.5	328.1414	1.1 × 10^5^	298.13 (100%); 289.12 (19%); 257.11 (12%) 208.60 (16%)	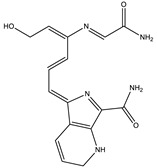	C_16_H_17_N_5_O_3_ Error = −3.7
10.5	655.2763	2.1 × 10^4^	637.26 (2%); 609.81 (1%); 596.26 (10%); 581.23 (2%); 551.23 (2%); 525.23 (1%) 445.04 (1%)	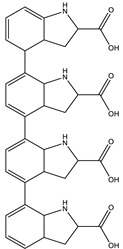	C_36_H_38_N_4_O_8_ Error = −0.15

**Table 2:** IC_50_ values for acetylcholinesterase (AChE) inhibition and EC_50_ values for DPPH scavenging activity of the *Pseudomonas putida* ESACB 191 and *Sepia officinalis* melanin, galantamine, butylated hydroxytoluene (BHT), and ascorbic acid. The standard deviation values were calculated based on three replicates for each condition; different superscript letters (a, b, c and d) correspond to values in the same assay condition that can be considered statistically different (95% confidence level). Not tested (-).

Samples	AChE	DPPH
	IC_50_ (µg/mL)	EC_50_ (µg/mL)
*P. putida* ESACB 191 melanin	575 ± 4.0 ^a^	74.2 ± 0.2 ^a^
*S. officinalis* melanin	1085 ± 8.7 ^b^	170.3 ± 0.5 ^b^
Galantamine	240 ± 6.0 ^c^	-
BHT	-	39.1 ± 0.3 ^c^
Ascorbic acid	-	4.4 ± 0.04 ^d^

**Table 3:** Cytotoxicity of the *Pseudomonas putida* ESACB 191 and *Sepia officinalis* melanins against A375 and HeLa Kyoto cell lines, IC_50_ values (mg/mL). The standard deviation values were calculated based on three replicates for each condition. Different superscript letters (a, b, c, d) correspond to values in the same assay condition that can be considered statistically different (95% confidence level). (-) not tested.

	A375	HeLa Kyoto	HepG2	Caco2
*Pseudomonas putida* ESACB 191 melanin	1.77 ± 0.09 ^a^	2.51 ± 0.06 ^b^	0.89 ± 0.14 ^d^	1.08 ± 0.09 ^d^
*Sepia officinalis* melanin	1.38 ± 0.03 ^a^	2.59 ± 0.05 ^c^	-	-

**Table 4:** *Pseudomonas putida* ESACB 191 melanin (1 mg/mL) and pharmaceutical compound ezetimibe (100 μM) in the inhibition of cholesterol (5 mM) permeation. The standard deviation values were calculated based on three replicates for each condition; different superscript letters (a, b, c) correspond to values in the same assay condition that can be considered statistically different (95% confidence level). (-) not tested.

	Inhibition of Cholesterol Permeation
	Basolateral (%)
Ezetimibe	87.5 ± 1.7 ^a^
Ezetimibe + melanin from *P. putida* ESACB 191	80.3 ± 1.2 ^b^
Melanin from *P. putida* ESACB 191	17.0 ± 4.2 ^c^

## Data Availability

Exclude the section no special data was reported in this section.
